# Examining gender diversity growth as a model for inclusion of all underrepresented persons in medical physics

**DOI:** 10.1002/mp.14524

**Published:** 2020-11-12

**Authors:** Maxine van Zyl, Elijah M. K. Haynes, Deidre Batchelar, Jennifer M. Jakobi

**Affiliations:** ^1^ Department of Psychology Faculty of Arts and Social Science University of British Columbia Okanagan 3333 University Way Kelowna BC V1V 1V7 Canada; ^2^ School of Health and Exercise Science Faculty of Health and Social Development University of British Columbia Okanagan 3333 University Way Kelowna BC V1V 1V7 Canada; ^3^ Department of Computer Science Mathematics, Physics and Statistics Faculty of Science University of British Columbia Okanagan 3333 University Way Kelowna BC V1V 1V7 Canada; ^4^ Department of Medical Physics BC Cancer – Kelowna 399 Royal Ave Kelowna BC V1Y 5L3 Canada

**Keywords:** climate in Medical Physics, gender diversity, inclusion, underrepresented persons

## Abstract

The labor force of Medical Physics is one of the most gender diverse in the field of Physics, as it has attained the proportional achievement of ~30% women worldwide (Tsapaki et al. *Phys Medica*. 2018;55:33–39). While great strides have been made toward a gender diverse workforce, women still comprise an underrepresented group. Many strategies have been suggested to increase the participation of underrepresented persons by addressing unconscious biases, increasing opportunities, dedicated hiring policies, and providing support networks in science and medicine (Barabino et al. *Sci Eng Ethics*. 2019; Coe et al. *Lancet*. 2019), yet the personnel landscape remains largely uniform. Herein, the conditions, strategies, and approaches that facilitated gender diversity in Medical Physics are considered as a means to further the inclusion of other underrepresented groups through exemplars of mentorship, addressing unconscious biases and the implementation of inclusive practices. Furthermore, the potential for gender diversity to act as a catalyst to create an environment that is more accepting of diversity and supports and encourages inclusive practices for the participation and inclusion of other underrepresented groups in Medical Physics is discussed.

## 1 INTRODUCTION

In the next 15 years, the global demand for Medical Physicists will continue to rise with an estimated 58 950 new Medical Physicists needing to be trained.^1^ To meet this demand, the field will be pushed to enlist a currently underutilized resource: underrepresented persons. These are defined as persons belonging to groups which make up a smaller percentage of a subgroup than their representation in the population. In Medical Physics, underrepresented groups include women, persons with disabilities, gender and sexuality minority (LGBTQ+) individuals, as well as racial and ethnic minorities. Diversity is categorized by variability (gender, age, sexuality, ethnicity) among individuals in a group, and inclusion refers to the degree to which individuals are integrated into a group, and allowed to contribute, as well as appreciated for their contribution. Increasing representation of underrepresented groups will require increasing both the diversity and inclusivity of Medical Physics.^2^, ^3^ Social justice, the notion that each individual within a society has a right to equal opportunities,^4^, ^5^ is inherent to diversifying Medical Physics; however, there are practical implications for both the field and healthcare generally. Diverse teams and organizations demonstrate improved productivity and creativity.^6^, ^7^ Additionally, superior financial gain has been observed for diverse organizations in the private sector.^8^ Fields that are homogenous tend to be exclusive, and experience stagnation and impaired clinical practice^9^, ^10^, ^11^, ^12^. In healthcare, there is growing awareness that specific concerns and practices of an underrepresented group (e.g., women) can be overlooked or misunderstood by bodies of clinicians and researchers. This may be due to a lack of community representation (diversity) in healthcare and research settings or a lack of cultural competency among providers, resulting in compromised healthcare service, and inappropriately narrow research.^13^, ^14^ These effects of lack of diversity can be observed by examining gender disparities in treatment techniques and effectiveness in pain management,^15^ oncology research,^16^ and physical and mental illness presentations.^13^

While Medical Physics is considered the most gender diverse field of Physics,^17^ it is far from being representative of the global population since only 29.8% (8702) of Medical Physicists worldwide are women.^1^ Women, and other underrepresented groups across the sciences, and within the field of Physics, report feeling excluded from social gatherings and meetings, not having their accomplishments and contributions recognized, and experiencing workplace discrimination and harassment.^10^, ^11^ Only 12% (US), 14% (Canada), and 18% (other countries combined) of female American Association of Physicists in Medicine (AAPM) members noted roles in clinical leadership^13^. Similarly, women were AAPM board seat‐holders, AAPM council chairs, Commission on Accreditation of Medical Physics Education Programs graduate or residency program directors, Medical Physics journal editors, or award winners in proportions lower than the overall percentage of female AAPM members.^18^ Furthermore, an examination of 10 million scientific papers in more than 6000 journals across Science, Technology, Engineering, Mathematics (STEM) and Medicine (STEMM) disciplines revealed a wide gender gap in author position, proportion, and presence in prestigious journals.^19^ In the field of Medical Physics, only ~22.5% of authors were women.^19^ Given that the proportion of female authors increases by 1% each year, Holman et al^19^ estimated that the field of Medical Physics may achieve gender parity in authorship in 25 years.

In addition to the lack of gender diversity, the race and ethnicity of Physicists is largely uniform. In 2017, only 13.5% of Bachelor, and 7.6% of Doctoral Physics degrees were awarded to underrepresented persons in the Unites States.^20^ Of the doctorate degrees awarded, 1 degree was awarded to a Native American, 19 to African Americans, and 62 to Hispanic or Latino individuals.^20^ Consequently, representation of racial and ethnic minorities in Physics faculties is severely lacking, with only 2.1% African American, 14.3% Asian, and 3.2% Hispanic members.^21^

To address a lack of diversity, some workplaces have set numerical targets for employing underrepresented persons. This practice may result in tokenism where individuals are hired for their demographic characteristics rather than skills and qualifications. This strategy to improve diversity should be avoided entirely for a few reasons. First and foremost, to deliver the highest level of healthcare possible, it is imperative that only competent individuals are involved in medicine and to do otherwise is unethical, and unfair to patients. Within organizations, the perception of tokenism often undermines the reputation of deservedly promoted individuals from underrepresented groups, as colleagues perceive them as undeserving. In contrast, some tokenistic hires are subsequently prevented from participation and advancement in the workplace^22^, ^23^ which reaffirms negative stereotypes about the competency of groups (e.g., women or people of color) to engage with technical subject matter (e.g., math and physics). Furthermore, stereotype threat, a form of social identity threat where an individual is anxious about confirming and being judged by stereotypes,^24^ is bolstered and contributes to a decline in individual performance, and in‐turn negative stereotypes about their group’s aptitude and abilities become self‐fulfilled. Individuals from underrepresented groups may also experience microaggressions (remarks or actions that are indirect, subtle, or unintentional discrimination^25^), due to emergent effects of unconscious biases, implicit assumptions, and ignorance held by other team members. Microaggressions can take the form of back‐handed compliments, condescending explanations (specifically, by a man to a woman; mansplaining^26^), underestimating competency, and unnecessary touching, which happens casually and frequently.^27^ These indignities serve to insult one’s group identity and sense of belonging^25^ and, although often unintentional, microaggressions impart a psychological, emotional, and physical toll upon recipients.^28^, ^29^ Additionally, these may occur within the context of intentional discriminatory actions or messaging such as name‐calling. Altogether, the above examples of exclusionary practices and behavior illustrate the fact that workplace diversity is ultimately meaningless when exclusionary behavior persists, and inclusion is not actively practiced.

Research suggests that diversity and inclusive practices within teams enhances the effectiveness of individual team members, and thus increases the output of the whole team.^6^, ^30^ These practices are not intended to reduce the availability of opportunities for majority group members. Rather, they are intended to improve the state of Medical Physics for all stakeholders. In fact, members of majority groups also benefit by interacting with co‐workers from backgrounds different from their own as this exposure to alternative perspectives promotes creative thinking and problem solving that contributes to improved team success.^6^, ^7^, ^31^ By keeping diversity and inclusive practices (e.g., gender inclusive hiring and evaluation policies; promoting psychological safety) in mind, and acknowledging the benefits thereof upon organizational output, previously untapped ingenuity is added and the overall state of Medical Physics as a field improves. In this work, we will first discuss how gender diversity can change the overall climate of the field, facilitating the inclusion of other underrepresented groups, and describe the benefits of diversifying the field. We will then examine the roles of mentors and educational outreach in increasing gender diversity and discuss the importance of retaining women in Medical Physics.

## 2 THE NEED FOR A DIVERSE MEDICAL PHYSICS WORKFORCE

While in the past clinical Medical Physicists may have spent the majority of their careers taking care of technology, somewhat in isolation from the rest of the care team, that is no longer the case. Increased treatment complexity and widespread development of automation for routine quality assurance have fundamentally altered how Medical Physicists impact patient care.^32^ Medical Physicists are an integral part of the interdisciplinary radiation oncology care team and frequently consult with Radiation Oncologists, Radiation Therapists, Medical Dosimetrists, as well as Nursing and other clinical fields, with their opinions informing clinical decisions. These fields may have significantly different gender balance and diversity compared to Medical Physics. While Nursing and Radiation Therapy remain female‐dominated fields, and while many medical specialties are approaching gender parity,^33^ Radiation Oncology's gender diversity is similar to Medical Physics. In 2015, 26.5% of American Radiation Oncologists were women, with the percentage of women increasing at 0.3% per year.^33^ Working in this context, increased diversity and inclusivity within Medical Physics, with its attendant improvements in communication and collective intelligence as outlined previously, can enhance relations with female dominated disciplines and the resulting overall warming climate may help to enhance overall diversity in Medical Physics.

Increasing diversity in the field of Medical Physics becomes more important as the field aims to further integrate into clinical practice. Medical Physics 3.0, the AAPM’s effort to provide a strategic direction for sustainable excellence and future growth for the field, has as an explicit goal for the profession that it expand beyond caring for technology into providing scientifically oriented healthcare as a specialist with direct relationships with patients.^34^ It is well established that diversity in healthcare professions allows for better patient care and outcomes.^35^ Thus, whether Medical Physicists are making technological choices for their clinics, helping their Radiation Oncology colleagues make treatment decisions, or interacting directly with patients to answer technical questions,^32^ diversity in Medical Physics will improve patient care. In turn, the contributions of Medical Physicists to patient care and outcomes become apparent.

Evaluating how diversity in medicine improves patient care provides a model by which diversity in Medical Physics might have parallel effects. Patient race and ethnicity influence the valence of a patient’s encounter with healthcare staff^36^, ^37^ when there is a lack of diversity, as differences in language and/or culture can give rise to misunderstanding or misinterpretation of patient concerns and behavior, physician treatment, and hospital protocols. This negative encounter is unique from prejudices and discrimination, and as important to change as eradicating racism. When ethnic background and language are matched between patients and healthcare staff, greater trust and understanding develops between them, thus contributing to patients exhibiting greater compliance with treatment regimes.^38^ While nonwhite physicians tend to serve within their own communities and other underserved populations (e.g., minority and foreign language patients), they remain underrepresented in healthcare teams.^39^ Therefore, by increasing the diversity of healthcare staff, underserved communities and individuals may receive better care by increasing the availability of representative healthcare staff within these communities.^39^ Furthermore, disparities between community and staff demographics can give rise to instances of incivility such as disrespectful comments and attitudes.^12^ However, when hospital staff are representative of the community they serve, civility toward patients improved, thereby contributing to higher quality care and positive experiences with healthcare that culminate in greater patient satisfaction.^12^ This demonstrates the need for a diverse workforce that is representative of the population it serves to ensure that patients not only receive optimal care, but also see semblances of themselves in the healthcare team contributing to positive experiences with healthcare professionals, and adherence to treatment recommendations.

As recognized by the AAPM MP3.0 Ad Hoc Committee,^32^ clinical medical physics' impact is not limited to direct clinical service. It also encompasses research directed toward clinical priorities. Diversity within research teams elevates awareness of potential research bias, and informs alternative approaches through the consideration of diverse viewpoints and expertise, which ultimately furthers innovation and clinical impact. Regarding gender diversity specifically, the presence of a female coauthor is positively associated with research studies investigating gender‐ and sex‐based differences in medicine.^40^ As gender‐ and sex‐related differences in treatment effectiveness have been historically downplayed,^13^ investigations into these concerns would improve healthcare and treatment options. Furthermore, an analysis of 9 million scientific publications and 6 million scientists, revealed that ethnic diversity in authorship was a significant predictor of scientific impact,^41^ providing additional motivation for leveraging gender diversity to increase overall diversity in Medical Physics.

While some may argue that diverse teams would be negatively impacted by conflict, diverse viewpoints, and personal background, this is not the case. A diverse workforce offers a distinctive lens of expertise which fosters productive differences, inclusive of professional conflict, that drives innovation.^42^, ^43^ Teams that have low relational conflict (interpersonal conflict due to differences in interpersonal style or taste) and high task conflict (related to opinions about the task at hand) are able to utilize conflict as a constructive and productive force to complete objectives.^42^ Additionally, it has been observed that gender‐diverse research and development teams exhibit increased innovation, culminating in novel designs.^7^

## 3 ADVANCING OTHER UNDERREPRESENTED GROUPS THROUGH GENDER DIVERSITY

Woolley et al.^6^ demonstrated that team performance on various tasks (deemed collective intelligence) was dependent upon the average social sensitivity among team members: an indicator of the team’s emotional intelligence. Those with high social sensitivity are more willing to collaborate and resolve conflict as well as respect conversational turn‐taking which improves productivity when collective intelligence is required.^30^ On average, women exhibit higher social sensitivity and perceptiveness than men,^44^ and a higher proportion of women resulted in greater emotional intelligence of the team.^6^ Joshi^45^ showed that women are more inclined to evaluate team members based on expertise and education, and therefore may be more adept at recognizing an individual’s potential for meaningful contribution to the group. Furthermore, teams with a higher proportion of women demonstrate stronger relationships and increased communication between members^31^ suggesting that the experience and knowledge of all team members can be better engaged when a critical number of women are involved.^30^ Thus, gender diversity, by the very fact that it inherently increases the number of women involved, enhances the aforementioned elements of communication, collaboration, conflict resolution, and engagement of all team members^6^, ^30^, ^31^ to the benefit of other underrepresented groups. Increasing the proportion of women across all levels of a team, forms a climate that encourages meaningful participation of all team members, irrespective of individual group membership.^31^, ^43^, ^45^

A key element in building successful teams and supporting collaborative efforts is psychological safety.^31^ This is defined as the belief that under no circumstances will you be humiliated or rejected for voicing opinions, asking for help or feedback, or for pointing out mistakes.^46^ Medical Physics relies on psychological safety as it relates to near‐miss events: situations where patient harm is narrowly averted. Reporting these events can serve as learning opportunities for all involved and identify procedural vulnerabilities, and risks to patients. Workplaces with higher degrees of psychological safety have a higher incidence of near‐miss event reporting, since individuals are able to report them and identify contributing factors without fear of social repercussions, thus contributing to a safer work environment and improved patient care.^47^ Inclusion and psychological safety begin by creating an environment where individuals feel respected and valued regardless of their personal characteristics and are encouraged to seize opportunities for contribution and engagement. This promotes the development of relationships and trust among team members, leading to closer knit teams.^30^ In exclusionary environments, trust and psychological safety cannot be fostered as unconscious biases, microaggressions, stereotype threat, and unfriendly work climates serve to cultivate distrust and division between members of minority and majority groups. Psychological safety allows individuals to direct their efforts toward problem solving rather than self‐preservation as individuals feel safe to express concerns, share ideas, and provide feedback without stereotype or social identity threat.^46^ In creating an environment that is supportive of inclusion and psychological safety, organizations can retain workers and encourage novel thinking.^48^, ^49^

Increasing gender diversity in Medical Physics would also disrupt exclusionary networks. In fields with greater gender balance, women report more support from all their peers as a network of relationships forms between all members of the group. In contrast, less diverse groups form relationships primarily between majority members, thereby excluding any remaining minority group members. Cain and Leahey^50^ demonstrate that when women are in the minority, they are excluded from informal relationship networks between colleagues, both in their immediate workplace and the broader scientific community. Informal relationships provide a supportive network, and often give rise to career development through research partnerships, speaking invitations, and social gatherings. Unfortunately, exclusion from these relationship networks hinders career development and integration into the workplace. However, when the proportion of women reaches a critical mass, these relationships become beneficial to all rather than harmful. In moving beyond token status to being integrated into the workplace, women can be connected to the network of relationships, enabling them to access a wide range of support systems and career development opportunities.^50^ A greater proportion of women and informal relationships also benefits other underrepresented groups, because the climate has been altered and the majority group curtailed by extension to include women. Thus, the continued expansion of these networks begins to embrace and include other underrepresented persons. Female leaders are also more likely to employ transformational leadership styles that further support inclusion and psychological safety by emphasizing the formation of relationships, nurturing the development of others through mentorship, and challenging the status quo to promote change.^51^ As gender diversity disrupts exclusionary networks and encourages the growth of inclusive networks, it promotes a safe and supportive atmosphere, softens potentially hostile environments, and assists underrepresented persons in connecting with team members and broader scientific communities.

## 4 EDUCATIONAL OUTREACH FOR INCREASING AWARENESS AND INTEREST IN MEDICAL PHYSICS

To achieve a gender diverse workforce, students (both men and women) need to first be attracted to Physics, then exposed to the field of Medical Physics and the varied career opportunities available therein, before graduate school. STEM outreach programs provide opportunities for early exposure to various fields and may bolster interest and achievement.^52^ While some Medical Physics outreach programs exist, they are less prolific than general STEM outreach programs. In addition to exposing students to the field, outreach programs also aid in supporting students during the transition from high school to undergraduate and graduate programs by ascertaining the importance of the field and illustrating personal value alignment within it. This will facilitate and enable continuance in the pursuit of STEM careers, inclusive of Medical Physics, despite challenges.^52^ Additionally, by educating youth from diverse backgrounds about the application of physics in medical research and practice, a wide range of potential trainees may be influenced to pursue Medical Physics as a profession, especially if they already intended to pursue a career in either medicine or Physics. As multiple intersections in patient care, innovation and impact exist between Medical Physics and medicine, candidates interested in both medicine and physics are able to combine their theoretical physics knowledge with the applications of medicine. While an emphasis should be placed on youth, outreach could also engage intergenerational populations, since knowledge and understanding of Medical Physics may be absent or limited in family and community members.^53^ Students who feel their chosen career path is supported by their families may also be more resilient to adversity later on.^54^

Similar to promoting within the field of Medical Physics to bolster recognition of accomplishments, social media platforms should be used to increase awareness of the professional health field. Social media is easily accessible, largely free, and widely used by individuals across a variety of age groups. Campaigns such as #ILookLikeAnEngineer have shown success in engaging individuals, organizations, and corporations in conversations about diversity in engineering.^55^ Similar methods could be employed to draw attention to Medical Physics as well as the varied individuals in the field and their accomplishments. Social media also provides a platform for like‐minded individuals to connect and access support networks worldwide. Connection mitigates feelings of isolation that come from being a minority, as well as encouraging collaboration and innovation.

Outreach emphasizing the communal qualities of Medical Physics may increase interest among girls and women who are drawn to helping professions. On average, women and girls are also drawn to fields and professions with a practical rather than a theoretical emphasis and those with a direct link to positive influence in society.^56^, ^57^ While the field of Medical Physics is focused on improving lives worldwide and has many practical applications, they are likely not well communicated in early education. By demonstrating the applications and impact of Medical Physics, a direct link to communal involvement and practicality can be made,^57^ drawing girls and women into the profession. Greater recruitment and retention enable a critical mass of female Medical Physicists to be achieved, thereby precipitating greater inclusivity for other underrepresented groups through the disruption of exclusionary networks, an increase in psychological safety, and overall shift toward an inclusive climate.^49^, ^50^

## 5 ROLE MODELS AND MENTORS: APPLICATION OF GENDER‐DIVERSITY SUCCESSES

Mentors and role models not only support the development of talent within and recruitment to the field but also aid in building a diverse and inclusive workplace.^58^ Although an individual can be both role model and mentor, each position serves a different purpose. Mentors actively support and guide mentees. Role models, on the other hand, enable individuals to envision themselves in a profession by providing opportunities for people to see characteristics of themselves in others who have attained success in said profession. Having role models for underrepresented persons frames the field as being inclusive of all individuals, thus increasing its appeal.

Observing the success of female Medical Physicists frames the profession as attainable for other women and fosters a sense of belonging.^59^, ^60^ In contrast, a lack of role models for women, or any other underrepresented group, engenders imposter syndrome whereby one’s individual success is perceived to be happenstance rather than due to aptitude and effort.^61^ By acknowledging the contributions of Medical Physicists from various underrepresented groups, role models become apparent to a diverse pool of potential future Medical Physicists who are passively encouraged to pursue similar career trajectories. Public awareness of such accomplishments can be achieved by highlighting research activities and other contributions to the field through, for example, social media, public lectures, or in postsecondary degree classroom settings. In this way young people are shown role models and diversity in the field. Such public awareness campaigns should use inclusive language and imagery when showcasing Medical Physicists. This can be achieved through respectful and balanced use of pronouns, including representations of women and different ethnicities in images, and simply referring to individuals as Medical Physicists without a descriptive suffix (i.e., female medical physicist). This last point may seem counter intuitive, but such descriptors focus attention on physical traits rather than professional designation. This will support perceptions of diversity within the field and emphasize opportunities for all. In doing so, more individuals will begin to view a successful career in medical physics as a viable career choice, thus increasing the pool of potential trainees and making that pool more diverse. In this manner, the growth of an inclusive climate is supported, the retention of individuals from uncommon backgrounds improves, and the field as a whole can benefit from diversity.

The visibility of female role models at various stages of career progression may also benefit the workforce as a whole. Women already established in their career benefit from role models, mentorship and mentoring as a means of engaging with their field, thus furthering their own careers and training the next generation.^2^ Role models provide evidence that it is not only possible for underrepresented group members to enter the field but also to excel and make contributions as leaders of organizations. They also demonstrate that obstacles specific to particular demographics can be overcome. For example, the retention of women and men in STEM fields is challenged by negative implications for a healthy family life.^62^, ^63^ Men also benefit from observing role models with healthy family/work life balance, as work is often overemphasized leading to mental and physical strain in men.^64^, ^65^, ^66^ Along these lines, female Medical Physicists in administrative and leadership positions with families can make excellent role models for younger physicists, both male and female; although, mentorship may be more effective if backgrounds are shared since majority group mentors may overlook obstacles faced by minority mentees.^67^ Matched mentors have often faced similar challenges as their mentees and may offer experiential targeted approaches to overcome these obstacles.^68^, ^69^ Additionally, there is evidence to suggest that women who are paired with woman mentors are more motivated and resilient than those without a mentor, or with a man as a mentor.^69^, ^70^ Having a positive mentee experience in biomedical sciences, may also encourage someone to mentor in the future.^68^ Thus, a sustainable network that supports retention of women and other members of underrepresented groups can naturally emerge from within the field of Medical Physics. The importance of mentorship to this end has been recognized by the AAPM, which offers a number of mentorship programs through its Professional Mentorship sub‐committee and the Science Council. Mentors and mentees can be paired through organizations such as Women in STEM and Engineering (WiSE) and scientific societies (e.g., the Canadian Organisation of Medical Physics), as well as through personal or professional connections. Organizations such as these can further support the growth of mentor–mentee networks by offering mentorship training events, awards, and advancement opportunities for mentors.

## 6 RETAINING AND SUPPORTING ESTABLISHED FEMALE MEDICAL PHYSICISTS

The strategies identified thus far have focused on increasing the number of women entering the field. However, retention of women already in the field is equally relevant, and perhaps more important since increasing the number of women entering Medical Physics will be ineffective at improving diversity if they are also leaving at a similar rate. Reasons for women leaving any field include experiencing unfriendly work environments and social isolation, as well as receiving less recognition for their achievements.^71^ Correcting overt wrongdoings and overlooked aspects such as minimizing the achievements and contributions of women (and other underrepresented persons), or ignoring them in formal conversations needs to be addressed by all persons involved (individuals, colleagues, and senior administration). Strategies for all cohorts are suggested in Table I to minimize exclusionary behavior within departments and teams, thereby providing individuals with an opportunity to contribute (see Table I; Woman, Interrupted), and be recognized for their work (see Table I; Authorship). Addressing unconscious biases and minimizing occurrences of microaggressions that contribute to stereotype threat can increase feelings of belonging which have been noted as a key component to women’s persistence in physical science fields.^60^ Research has demonstrated that strong stereotypes about the gender of a scientist (gender‐science stereotype) exist in nations where men dominate scientific fields and are in favour of viewing scientists as men; even when the nation also exhibits high gender equity.^72^ However, in nations where there is high female participation in science fields, the gender‐science stereotype is weaker. This weakened gender‐science stereotype could be because increased awareness of women in science occurs when a greater proportion of women are present in these fields.^72^

**Table I:** Examples of situations and approaches to enhancing the workplace environment to facilitate inclusivity and diversity in the Medical Physics discipline.

Woman, Interrupted		
At an interdepartmental meeting of Radiation Oncologists, Radiation Therapists, and Medical Physicists, the Chief RO asks for the opinions of the MPs on a matter. He asks for the opinions of two male MPs by name and ignores the lone female MP. In addressing the Chief RO’s question, male MP1 asks for the opinion of the female MP. While providing her recommendation, she is promptly interrupted by male MP2, who reiterates what the female MP had said and is subsequently praised for his input.		
*Actionable Steps*		
Female MP	Chief RO	Bystander(s)
1 Be aware of what is occurring. 2 Regularly and actively participate in discussions to establish herself as a contributing member 3 Politely interrupt male MP2 and reiterate her opinion 4 If being ignored and interrupted is a common occurrence, raise the issue with team leaders 5 Seek out mentors who can provide support and guidance on how to address these concerns	1 Should lead by example by allowing all team members to participate equally in discussions and receive acknowledgment for their contributions. 2 Encourage participation and create space for team to provide their input and raise concerns, without interruptions. 3 Consider establishing protocols to hear all persons’ opinion.	1 The Chief MP should meet with the Chief RO, gather the team and openly acknowledge that this behavior is exclusionary 2 Interrupt the interrupter to allow the female MP to continue 3 Follow male MP1’s lead in redirecting questions to the female MP 4 A senior female/male staff member could mentor the female MP and support her in actively contributing to discussions and raising awareness of exclusionary actions
Authorship		
In a large mixed‐gender research laboratory of graduate students, research assistants, and postdoctoral fellows (PDF) collaboration across projects occurs frequently. Male graduate students and PDF are added as middle authors to others’ manuscripts while female graduate students and PDF are merely mentioned in the acknowledgments. The explanation given is that the nature of the females contributions did not meet the requirements for authorship.		
*Actionable steps*		
Female students	Male students	Laboratory members
1 Support one another by giving credit where it is due 2 Address the issue and occurrences with the lead scientist. Have evidence and provide observation based on fact.	1 Acknowledge the contributions of female students and advocate for equal privileges to be awarded to them 2 Support them and address the issue with the lead scientist	1 Discuss authorship at the early stages of the project and during if roles and responsibilities evolve 2 Identify clear and concrete requirements for authorship (e.g., level of contribution, intellectual thought) 3 Outline specific tasks accomplished by each individual to highlight contributions 4 Determine whether contributions to a project meet the requirements of authorship through peer‐evaluations and group consensus
The Hug		
On his last day of work before the holidays, the male Chief Physicist gives handshakes to the other men while wishing them Merry Christmas and Happy New Year. For the only woman and most junior member in the group, he gives a hug. She is clearly uncomfortable being singled out.		
*Actionable steps*		
Junior member	Chief physicist	Bystander(s)/organization/team
1 Speak with the Chief Physicist or his supervisor should the behavior continue 2 Start a conversation on whether/when hugging in the workplace is acceptable.	1 If unaware of the impact of his actions, apologize upon identification from others. 2 Lead by example. Awkward hugs are common; acknowledge it, apologize and avoid a repeat occurrence. 3 Recognize cultural approaches to holiday greetings	1 If comfortable, interrupt the hug. There may also be an opportunity for witty remarks such as “why didn’t I get a hug?.” This may serve to normalize hugging between all members of the team to avoid singling members out, or halt any future hugs 2 Speak with the Junior Member about her experience and offer support 3 Speak to the Chief Physicist about his actions and their impact 4 View this conflict as an opportunity for change in the workplace; introduce holiday practices and policies
Parental leave		
“Since my spouse returned from leave, our manager avoids asking her to stay after‐hours for work that is normally her responsibility. For example, once he finds out about repairs that need to be done on her equipment, or that there is an emergency patient coming in that needs to be treated in the evening, he stays late instead of her. Generally, no one wants to do after‐hours work, but it is a responsibility we all have at one time or another and she shouldn’t have to fight for tasks that are her responsibility. He thinks he’s being helpful, but his actions highlight the differences between our colleagues and her.”		
*Actionable steps*		
Returning spouse	Concerned spouse	Manager/team/organization
1 Practice self‐affirmation: the singling out is due to the stereotype (i.e., that she needs a lighter workload), not her aptitude 2 Identify other potential reasons for reducing her workload 3 Stay late and avoid the perception of rushing out the door	1 Support spouse by coordinating childcare for days when she has to or previously would have stayed late	1 The manager should consult the returning spouse before making decisions regarding her availability, and identify any new policies that may influence his decision 2 A senior female staff member can act as a mentor by modeling best practices for a balanced work/home life and meeting with the returning spouse to develop an action plan 3 Actively include the returning spouse in discussions and decision making in the workplace, as well as inviting them to participate in after hours events (e.g., gathering for drinks) or creating lunch/daytime events to mitigate feelings of isolation or exclusion 4 Consider adding on‐site childcare 5 Scheduling flexibility and support of work/life balance across the whole team
Cultural insensitivity		
One member of a team is an observant Hindu who does not eat beef. The Chief Physicist buys lunch for the group and chooses dishes that mostly contain beef. When the food arrives, the Chief Physicist tells the observant Hindu to simply pick the pieces of beef out of the dish.		
*Actionable steps*		
Observant hindu	Chief physicist	Bystander(s)/organization
1 Start a conversation on cultural and religious practises to educate the organization/team as well as provide an opportunity for others to share their practices. 2 Suggest recipes, dishes or restaurants that cater to many dietary restrictions	1 Openly apologize to the Hindu team member. 2 In the future, request team members privately disclose dietary restrictions	1 Identify to the Chief Physicist that his suggestion was unsatisfactory 2 As a team, recognize that instances of cultural insensitivity may occur unintentionally, and discuss potential protocols and/or approaches to address and avoid such instances 3 Suggest recipes, dishes or restaurants that cater to dietary restrictions
Examples and suggestions presented herein are not exhaustive, and situational context afford unique approaches. These cases serve to exemplify that change arises from all individuals. Supporting the growth of an environment rich in communication, understanding, and respect of all persons and opinions is for the betterment of the team and its members and will ultimately develop an inclusive climate.		

Additionally, when policies and procedures are combined with inclusive practices the outcome successes are greater in reducing sexual harassment, lessening tokenism, and in actively supporting and enhancing the participation and integration of women in the workplace. Rather than being relied on to produce an inclusive climate, these policies should work alongside the organizational culture by serving as a reminder of the organization’s dedication to implementing inclusive practices and fostering a respectful climate for all employees.^3^ When organizational culture or employee behavior is contrary to these practises, they are not effective at reducing harassment, or ensuring the safety and comfort of employees. Hall et al.^73^ noted that gender‐inclusive policies were more predictive of lower social identity threat when women had more positive experiences with male colleagues. This finding demonstrates the role of culture as a mediating factor between policy and practice, and the incidence of social identity threat. Additionally, while sexual harassment policies are widely employed, a survey of female undergraduate physics students revealed that 74.3% of respondents had experienced sexual harassment in a physics environment.^74^ Sexual harassment influences one’s sense of belonging, thus decreasing intentions to persist in the field.^60^, ^74^ Furthermore, simply educating individuals about sexual harassment tends to increase incidences and backlash, with similar results noted in diversity training.^75^ Therefore, it is crucial that the underlying culture is addressed, and as identified in Table I through the example of “The Hug,” it is imperative that all individuals be part of the solution to ensure the overall climate is appropriately inclusive, rather than relying solely on policies to maintain a respectful environment. Change at the institutional level includes supporting gender‐inclusive policies, language and imagery, and anonymizing evaluation procedures to reduce stereotype threat.^76^

Furthermore, workplaces that allow conscious or unconscious biases and microaggressions to continue unchecked are unwelcoming, and prompt feelings of isolation and incompetence, contributing to an exclusionary environment and resulting in individuals leaving the workplace and/or field.^60^ In this fashion, exclusionary behaviors and environments serve to discourage underrepresented groups from both entering and persisting in Medical Physics by contributing to an overall chilly climate.^10^, ^11^ Appeal of the field and persistence therein are mediated by climate, further illustrating the need to curb exclusionary practices contributing to cooler climates which lessen diversity. These climates might be unintentionally created through best intentions to support individuals, and this is often observed through supportive leadership during periods of health crisis and return to work (see Table I; Parental Leave). Minimizing the impact of unintentional segregation as well as personal unconscious biases and microaggressions can be facilitated by undergoing training to deconstruct biases through exposure to individuals who oppose preconceived stereotypes and identifying similarities between oneself and individuals of other groups.^77^ This also extends to biases surrounding cultural practices and religion, and friction that may arise due to misunderstanding or lack of understanding (see Table I; Cultural Insensitivity). It is imperative to note that singular unconscious bias training events will not facilitate reductions in unconscious bias, rather active decisions to alter responses and thinking mediate the impact of unconscious biases.^77^

It is further recommended that organizational policies include flexible scheduling to accommodate childcare needs, for both men and women.^78^ Institutions should also consider providing funding for further education and financial supplements that assist women in career development (e.g., attending leadership workshops and conferences). Such initiatives advance the perception of women in the field as well as facilitate networking opportunities which further minimizes isolation factors that limit diversity in Medical Physics. Yet another key consideration for advancing gender diversity and supporting inclusion is career tracks for women to work toward leadership positions. This supports the integration of women into the field and enables accomplished women to become more visible as role models for young women entering Medical Physics.^54^, ^79^

## 7 CONCLUSION

Ultimately, diversity and inclusion work in tandem. Meeting diversity quotas will not realize the fruits of diversity if inclusive behavior is not present. Diversity quotas may provide opportunities for women to enter the workforce, but may also encourage tokenism, leading to alienation and loss of female employees. Inclusive practices should provide everyone the opportunity to be a contributing member and be valued for their opinion and accomplishments. Every marginalized group faces a unique set of challenges in the pursuit of proportional representation in Medical Physics. As such, the specific strategies to improving the representation of each group are required to be unique. Regardless of which group is underrepresented in any given setting, the practice and promotion of inclusivity is central to any one of these strategies. In its absence, quantifiable improvements to diversity in medical physics are merely performative at best. While achieving maximum heterogeneity in medical physics is certainly an intergenerational challenge, small steps can be taken today to make incremental improvements in the field. By creating an inclusive environment for women in medical physics, inclusive practices can be established and generalized to improve inclusivity for other underrepresented groups. By increasing the representation and inclusion of women, gender diversity can act as a catalyst for the development of an atmosphere that is accepting of other forms of diversity (e.g., age or ethnicity) and actively strives to encourage the participation of all underrepresented persons. A diverse and inclusive environment in Medical Physics will allow for greater innovation,^7^ more applicable research and improved healthcare^43^ as well as drawing individuals to the field. As such, gender diversity, and the approaches that have allowed it to develop, can be viewed as a model to grow a diverse community in the field of Medical Physics.

## AUTHORS' CONTRIBUTION

M.v.Z. and E.H. developed the manuscript. J.J. and D.B. revised and made amendments throughout manuscript development. The final submission copy was approved by all four authors.

## CONFLICT OF INTEREST

The authors have no conflict to disclose.
